# Pentosan Polysulfate Decreases Myocardial Expression of the Extracellular Matrix Enzyme ADAMTS4 and Improves Cardiac Function *In Vivo* in Rats Subjected to Pressure Overload by Aortic Banding

**DOI:** 10.1371/journal.pone.0089621

**Published:** 2014-03-03

**Authors:** Maria Vistnes, Jan Magnus Aronsen, Ida G. Lunde, Ivar Sjaastad, Cathrine R. Carlson, Geir Christensen

**Affiliations:** 1 Institute for Experimental Medical Research, Oslo University Hospital and University of Oslo, Oslo, Norway; 2 KG Jebsen Cardiac Research Center and Center for Heart Failure Research, University of Oslo, Oslo, Norway; 3 Bjørknes College, Oslo, Norway; 4 Department of Genetics, Harvard Medical School, Boston, Massachusetts, United States of America; Albert Einstein College of Medicine, United States of America

## Abstract

**Background:**

We hypothesized that cleavage of the extracellular matrix (ECM) proteoglycans versican and aggrecan by ADAMTS (a disintegrin and metalloprotease with thrombospondin motifs) proteases, which contributes to stress-induced ECM-reorganization in atherogenesis and osteoarthritis, also play a role in heart failure development.

**Objectives:**

The primary objective was to identify alterations in expression of ADAMTS versicanases and aggrecanases during development of heart failure, while evaluation of the effects of *in vivo* modulation of relevant changes in ADAMTS activity constituted the secondary objective.

**Methods:**

Myocardial levels of versican, aggrecan, and their ADAMTS cleaving proteases were examined in Wistar rats six weeks after aortic banding (AB), and versican and selected ADAMTS versicanases were further analyzed in neonatal cardiomyocytes (NCM) and cardiac fibroblasts (NFB) after stimulation by inflammatory mediators. Based on the initial findings, ADAMTS4 was selected the most promising therapeutic target. Thus, rats with AB were treated with pentosan polysulfate (PPS), a polysaccharide with known ADAMTS4-inhibitory properties, and effects on versican fragmentation, left ventricular function and geometry were evaluated.

**Results:**

We discovered that myocardial mRNA and protein levels of ADAMTS1 and -4, and mRNA levels of versican, aggrecan, and ADAMTS8 increased after AB, and TNF-α and IL-1β synergistically increased mRNA of versican and ADAMTS4 in NCM and NFB and secretion of ADAMTS4 from NCM. Furthermore, PPS-treatment improved systolic function, demonstrated by an improved fractional shortening (vehicle 48±3% versus PPS 60±1%, p<0.01) after AB. Following PPS-treatment, we observed an ∼80% reduction in myocardial ADAMTS4 mRNA (p = 0.03), and ∼50% reduction in the extracellular amount of the p150 versican fragments (p = 0.05), suggesting reduced versicanase activity.

**Conclusions:**

Our findings suggest that AB induces an increase in myocardial ADAMTS4 versicanase activity, and that PPS-treatment improved systolic function in the pressure-overloaded heart, holding promise as a novel therapeutic agent in heart failure.

## Introduction

The fatal development of remodeling and failure of the pressure-overloaded heart is insufficiently prevented by today's therapy. Extracellular matrix (ECM) reorganization is gaining recognition as an important contributor to heart failure progression [1], and inhibitors of matrix metalloproteases (MMP) modulating collagen turnover have been extensively studied in the search for novel and effective heart failure drugs [2]. However, promising pre-clinical results have not been translated into clinical benefits [3]. Thus, other ECM components should be evaluated as targets for heart failure therapy, and the family of proteoglycans, where some members are associated with heart failure development [4], [5], represent potential candidates.

Although it has been shown that the large hydrophilic proteoglycans versican and aggrecan reside in the embryonic cardiac ECM [6], and that versican is expressed in cardiomyocytes [7] and the adult heart [8], their role in heart failure development is unknown. In other organs, versican and aggrecan are important regulators of ECM volume and hydration due to their large content of water-binding glucosaminoglycan (GAG) chains, and their ability to aggregate with the GAG molecule hyaluronic acid (HA) [9]. The degradation of versican and aggrecan by ADAMTS (a disintegrin and metalloprotease with thrombospondin motifs) proteases is essential in ECM reorganization in both pathological and physiological processes, including osteoarthritis, atherosclerosis, and regulation of cardiac organogenesis [10]–[13]. Interestingly, ADAMTS4 synthesis in macrophages increases in response to tumor necrosis factor (TNF)-α and interleukin (IL)-1β [14], cytokines which are found increased in the myocardium of heart failure patients [15], [16], suggesting a role in heart failure development.

The primary objective of the study was to identify alterations in expression of ADAMTS versicanases and aggrecanases during development of heart failure. Myocardial levels of versican, aggrecan, and their cleaving ADAMTS proteases were examined in rats exposed to aortic banding (AB), subdivided into groups with preserved (HFpFS) or reduced (HFrFS) fractional shortening (FS). Versican and selected ADAMTS proteases were further analyzed in neonatal cardiomyocytes (NCM) and cardiac fibroblasts (NFB) after treatment with inflammatory mediators. The secondary objective of the study was to evaluate the effects of *in vivo* modulation of relevant changes in ADAMTS activity. Based on the initial findings, we identified ADAMTS4 as the most promising target among the ADAMTS proteases. Therefore, rats with AB were treated with pentosan polysulfate (PPS), a polysaccharide with known ADAMTS4-inhibitory properties [17], [18], and effects on heart function, versican expression level and fragmentation were studied.

## Materials and Methods

### Ethics statement

The experimental procedures conformed to the *European Convention for the Protection of Vertebrate Animals Used for Experimental and Other Scientific Purposes*, and are reported according to the ARRIVE guidelines. The protocols were approved by the Norwegian Council for Animal Research prior to the beginning of the study. All surgical procedures were performed in full anesthesia by an investigator with extensive experience in rodent experimental models (JMA), and all efforts were made to minimize suffering.

### Animal model

Male Wistar rats weighing 160–170 grams (approximately 7 weeks old) (Taconic, Skensved Denmark) underwent AB essentially as described previously [19]. Briefly, the ascending aorta was dissected free through a right hemithoracotomy, and ligated (3-0 silk) against a steel wire. The sham-operated rats were subjected to the same surgical procedure with a loose suture around the aorta. In all surgical procedures performed, a mixture of 67% N_2_O, 28% O_2_, and 4% isoflurane in an anesthesia chamber was used for preoperative sedation. To maintain anesthesia peroperatively, a mixture of 69% N_2_O, 29% O_2_, and 2% isoflurane was given by the endotracheal tube and the animals were ventilated on a respirator (Zoovent, Triumph Technical Services, Milton Keynes, UK). Buprenorphine (0.2 mg/kg) was given as postoperative analgesia after AB. Rats were housed in cages (two rats in each cage) with Bee Kay bedding (Scanbur BK, Nittedal, Norway) in 55% humidity on a 12 h light/dark cycle, with food pellets (RM1, 801151, Scanbur BK) and water ad libitum.

### Animal phenotyping and administration of PPS in AB rat model

In vivo heart function was evaluated using the Vevo2100 system (Visualsonics Inc, Canada) as previously described [20]. Thereafter, the heart was excised under deep anesthesia, washed in saline and blotted dry to remove blood in the cardiac chambers. The heart chambers were separated by rapid dissection, before left ventricles were snap-frozen in liquid nitrogen and stored at −70°C until RNA and protein analyses.

In the first part of the study, myocardial samples were harvested six weeks after induction of AB, and rats were divided into subgroups with preserved (HFpFS) or reduced FS (HFrFS), defined as FS above or below 50% based on findings from previous studies [19] Inclusion criteria were based on echocardiographic and post-mortem analysis as follows: Inclusion criteria for HFrFS group were increased lung weight (>2.5 g), increased posterior wall thickness at diastole (>2.0 mm), and increased left atrial diameter (>5.0 mm), while criteria for inclusion in the HFpFS group were increased posterior wall thickness at diastole (>2.0 mm), increased left ventricular weight (>0.75 g), and preserved lung weight (<2.0 g). Animals not satisfying inclusion criteria were excluded from the study. Based on power analysis, inclusion of minimum six rats in each group could detect a ≥2-fold increase in mRNA levels (power 0.8, α = 0.05).

For administration of PPS, rats were stratified to an intervention or control group, pairing two and two rats with similar gradient over the stenosis measured at post-operative day 3, in order to ensure similar degree of cardiac stress. The intervention group was treated with 6 mg/kg of sodium PPS (Interfarm AS, Norway), while a control group received vehicle (0.9% NaCl), both injected subcutaneously every third day. The dosage was selected based on previous experimental studies exploring other effects of PPS-treatment [21]–[23], and recommended dosage regimens for human and veterinary medicine assuming a bio-availability of PPS close to heparin for subcutaneous injections. Rats were observed daily and no side effects including hemorrhage were observed. Six weeks after AB or sham operation, rats were examined by echocardiography and post-mortem analysis, performed by experienced investigators (IS, JMA) blinded to treatment group. In addition, mRNA and protein analyses on myocardial tissue were performed blinded to treatment group. Power analysis revealed that a minimum of 14 rats in each AB-group could detect a ≥8% increase in FS in rats receiving PPS-treatment compared to vehicle-treatment (power 0.8, α = 0.05).

### RNA isolation, reverse transcription and qRT-PCR

The mRNA levels of ADAMTS1, -4, -5, and -20, possessing versicanase and/or aggrecanase activity and ADAMTS8, -9, -15, -16, and -18 possessing aggrecanase activity [24] were quantified in rat left ventricle by qRT-PCR. ADAMTS demonstrating altered mRNA levels after AB were also quantified after PPS-treatment. In addition, mRNA levels of proteins known to modulate ADAMTS activity or versican function were measured; tissue inhibitor metalloprotease (TIMP)-3, HA synthase-1 and -2, and membrane-type (MT)4-MMP, while mRNA levels of versican and the versicanases ADAMTS1 and -4 were quantified in NFB and NCM.

Total RNA was isolated from the left ventricle in AB rats, rat NCM and NFB (RNeasy mini kit, Qiagen, Valencia, CA). All RNA samples were quality assessed, with RNA integrity numbers (RIN)>7.5 (Agilent Bioanalyzer, Agilent Technologies, Palo Alto, CA, USA) and 280/260 ratios >2 (Nanodrop ND-1000 Spectrophotometer (Thermo Scientific, IL) accepted. Reverse transcription was performed with iScript Select cDNA Synthesis Kit (Bio-Rad Laboratories, Inc., Hercules, CA). Pre-designed TaqMan assays (Applied Biosystems, Foster City, CA) were used in quantitative real-time polymerase chain reaction (qPCR) to determine gene expression of ADAMTS1 (Rn01646120_g1), ADAMTS4 (Rn02103282_s1), ADAMTS5 (Rn01458486_m1), ADAMTS8 (Rn01524921_m1), ADAMTS9 (Rn01425216_m1), ADAMTS15 (Rn01524703_m1), ADAMTS16 (Rn01537448_m1), ADAMTS18 (Rn01426916_m1), ADAMTS20 (Rn01407540_m1), versican (Rn01493755_m1), aggrecan (Rn00573424_m1), HAS-1 (Rn01455687_g1), HAS-2 (Rn00565774_m1), TIMP-3 (Rn00441826_m1) and MT4-MMP (Mm00449292_m1). For normalization, the reference gene ribosomal protein L4 (RPL4) (Rn008211091_g1) was used. Results were detected on a 7900HT Fast Real Time PCR System (Applied Biosystems, CA).

### Protein isolation

Frozen left ventricle from the HFpFS , HFrFS and corresponding sham group, was pulverized in a mortar with liquid nitrogen. Ice cold lysis buffer (20 mM Hepes pH 7.5, 150 mM NaCl, 1 mM EDTA, 0.5% Triton X-100) with protease inhibitors (Complete EDTA-free tablets, Roche Diagnostics, Germany) was added and the samples homogenized for 3×1 minutes with a Polytron 1200 (Capitol Scientific, Austin, TX), incubated on ice for 30 minutes and centrifuged at 70 000 *g* for 60 minutes at 4°C. The supernatants were stored at −70°.

### Fractional protein isolation

Fractional protein lysates from myocardium of rats with AB treated with PPS or vehicle were extracted as previously described [25]. Briefly, proteins were separated into three fractions; NaCl-soluble extracellular proteins (NaCl-ECM) and guanidine-soluble extracellular proteins (G-ECM), containing the fluid and matrix compartment of ECM respectively, in addition to cellular and cell-linked proteins soluble in SDS (SDS-samples). NaCl-ECM and G-ECM were deglycosylated using 4 mU/NaCl-sample and 12 mU/G-sample of Chondroitinase ABC (from Proteus vulgaris, G3667, Sigma-Aldrich, MO) and 5 mU/NaCl-sample and 15 mU/G-sample of keratanase (endo-β-galactosidase from Bacteroides fragilis, G6920, Sigma-Aldrich), essentially as previously described [25].

### Immunoblotting

Protein concentrations were measured using the Micro BCA Protein Assay Kit (Pierce/Thermo Scientific, IL). Sample buffer was mixed with 30 µg (NaCl and SDS samples) or 5–25 µg (G-samples) of the protein lysate, denatured at 96°C for 10 min, and run on 1.5 mm 4–12% Bis-Tris gradient gels (Nupage, Invitrogen, CA), with MOPS SDS running buffer until the 37 kDa protein marker reached the bottom of the gel. All protein extracts were loaded blinded to group.

SDS-PAGE and immunoblotting was performed as described in the Criterion BioRad protocol, using polyvinylidene fluoride (PVDF) membranes (GE Healthcare Life Sciences, Uppsala, Sweden). Blots were developed using the ECL Plus Western Blotting Detection System (GE Healthcare), visualized in the Las-4000 mini from Fujifilm (Japan) and quantified using ImageJ software (NIH). Before reprobing, stripping was performed using the Restore Western Blot Stripping Buffer (21059, Thermo Scientific, IL).

Coomassie staining of the blots was chosen to control for equal loading in fractional protein lysates, while anti-glyceraldehyde 3-phosphate dehydrogenase (GAPDH) (sc-20357, Santa Cruz Biotechnology) was used as loading control for protein lysates from HFpFS, HFrFS and the corresponding sham group. Equal loading was observed for GAPDH, and for Coomassie for SDS and NaCl protein fraction samples. For G-fractions, Coomassie staining revealed signs of potential unequal loading, thus protein levels in these samples were normalized to the 65 kDa band in the corresponding Coomassie stained blot.

### Antibodies

Primary antibody used was versican fragment displaying the neo-epitope DPEEAE resulting from cleavage by ADAMTS1/4 [26] (ab19345, Abcam, Cambrigde, UK) or ADAMTS4 (PAI-175, Thermo Scientific, IL), and secondary antibody used was goat anti-rabbit IgG-HRP (4030-05, Southern Biotech, AL), diluted in 1% casein (versican DPEEAE and secondary antibody) or 5% non-fat dry-milk (170-6404, BioRad) (ADAMTS4). Membranes were blocked in 1% casein for one hour in room temperature, incubated overnight at 4°C with primary antibody, and for one hour in room temperature with secondary antibody.

### Neonatal cardiomyocytes and fibroblasts

Primary neonatal cardiomyocytes (NCM) and fibroblasts (NFB) were isolated from 1–3 day old Wistar rats (Taconic, Skensved, Denmark) as previously described [5]. Briefly, hearts were removed and the left ventricle dissected and digested using collagenase, and cell suspension was transferred to uncoated culture flasks allowing NFB to attach, while NCM were transferred to gelatin-coated culture dishes (Corning International, Corning, NY) in plating medium [Dulbecco's Modified Eagles Medium (Sigma) supplemented with penicillin/streptomycin (Sigma), 44 mmol l^−1^ NaHCO, medium 199 (Sigma), 26 mmol l^−1^ NaHCO3, horse serum (14-703E, Bio-Whittaker,Walkersville, ML) and fetal calf serum (14-701E, Bio-Whittaker)]. Cardiac cells were starved in serum-free medium (plating medium without serum) 24 hours before treatment with 10 ng/ml IL-1β (501-RL-010, R&D Systems, Minneapolis, MN) and 50 ng/ml TNF-α (PMC3014, BioSource International, Camarillo, CA) separately and combined, and 1000 ng/ml lipopolysaccharide (LPS) (List laboratories, Campbell, CA) for 24 h. Cell medium was ultrafiltrated using Amicon Ultra-4 10 kDa centrifugal filter (Millipore, MA) before protein analyses.

### Statistical analyses

Depending on the distribution, data are reported as mean±SEM or median (interquartile range) and comparisons between groups investigated with Student's *t*-test or Mann-Whitney test. Mortality rates were compared using Pearson's Chi Square test. P-values (two-sided) ≤0.05 were considered significant. Dunn's correction for multiple comparisons was used for cell culture mRNA data. Data are normalized to RPL4 and control (mRNA) or control (protein).

## Results

### Myocardial expression of ADAMTS versicanases is increased in rats after AB

**Figure 1:**
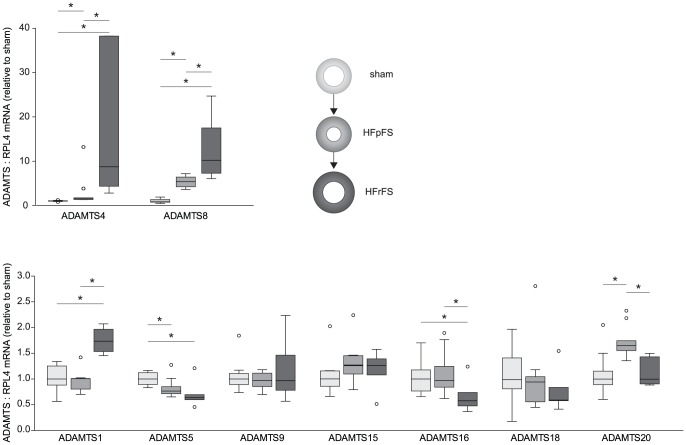
Elevated mRNA levels of ADAMTS1, -4 and-8 in rats after aortic banding. Myocardial mRNA expression of ADAMTS versicanases and aggrecanases in rats with preserved (HFpFS, n = 9) or reduced FS (HFrFS, n = 6) six weeks after AB, normalized to reference gene RPL4 and relative to sham (n = 9). Box-plots show median (horizontal line), interquartile range (box), 1.5xinterquartile range or maximum/minimum range (whiskers) and outliers (>1.5xinterquartile range). *p<0.05. AB, aortic banding; FS, fractional shortening; ribosomal protein L4, RPL4.

Myocardial mRNA levels of ADAMTS proteases with known versican and/or aggrecan activity were measured in the two subgroups of AB-rats (animal characteristics are given in Table 1). In addition to included rats, 3 rats died before reaching post-operative week 6. ADAMTS1, and -4, with known versicanase and aggrecanase activity, and ADAMTS8 with known aggrecanase activity, demonstrated enhanced mRNA levels in AB-rats with reduced FS. More specific, ADAMTS1 mRNA level was 1.7-fold higher in HFrFS (1.74 (1.51–1.99)) than in both HFpFS (1.00 (0.80–1.01), p<0.01) and sham (1.00 (0.80–1.30), p<0.01), while ADAMTS4 mRNA level in HFrFS (8.75 (3.97–38.21), p<0.01) was ∼9-fold higher than sham (1.00 (0.95–1.11), p<0.01) and 5-fold higher than HFpFS (1.65 (1.44–2.88), p = 0.01). Similarly, mRNA levels of ADAMTS8 mRNA in HFrFS (10.22 (7.00–19.36) were ∼10-fold higher than sham (1.00 (0.66–1.53), p<0.01) and ∼2-fold higher than HFpFS (5.44 (4.04–6.64), p<0.01) (Figure 1).

**Table 1:** Echocardiographic analyses and organ weights in AB-rats.

	Sham	HFpFS	*Sham vs HFpFS (p-value)*	HFrFS	*Sham vs HFrFS (p-value)*	*HFpFS vs HFrFS (p-value)*
N	9	9		6		
ORGAN WEIGHTS						
Body (g)	367±6	330±14	*0.11*	329±12	***0.01***	*0.65*
Heart (g)	0.99±0.03	1.59 ± 0.07	***<0.01***	2.77±0.13	***<0.01***	***<0.01***
LV (g)	0.52±0.02	0.96±0.04	***<0.01***	1.34±0.06	***<0.01***	***<0.01***
RV (g)	0.15±0.01	0.15±0.01	*1.00*	0.33±0.04	***<0.01***	***<0.01***
Lung (g)	1.05±0.07	1.56±0.07	***<0.01***	3.82±0.19	***<0.01***	***<0.01***
M-MODE LV						
IVSd (mm)	1.46±0.04	2.37±0.10	***<0.01***	2.47±0.03	***<0.01***	*0.46*
LVDd (mm)	6.25±0.14	5.71±0.20	***0.04***	7.71±0.29	***<0.01***	***<0.01***
LVDs (mm)	2.69±0.16	2.17±0.13	***0.03***	5.00±0.25	***<0.01***	***<0.01***
FS (%)	57±2	62±2	*0.10*	35±2	***<0.01***	***<0.01***
PWd (mm)	1.62±0.04	2.42±0.07	***<0.01***	2.54±0.05	***<0.01***	*0.20*
M-MODE AO/LA						
LAD (mm)	3.42±0.06	4.32±0.08	***<0.01***	6.57±0.37	***<0.01***	***<0.01***
Aorta (mm)	2.53±0.04	2.56±0.03	*0.56*	2.58±0.05	*0.43*	*0.73*
DOPPLER						
Peak mitral flow (m/s)	834±34	854±42	*0.71*	1094±63	***<0.01***	***<0.01***
Mitral deceleration (m/s)	2368±149	3288±236	***<0.01***	5405±437	***<0.01***	***<0.01***
Heart rate	414±8	420±9	*0.65*	378±7	***<0.01***	***<0.01***
TISSUE DOPPLER						
Maximal systolic velocity	77±3	52±2	***<0.01***	31±1	***<0.01***	***<0.01***
Maximal diastolic velocity	85±3	−43±10	***<0.01***	−49±3	***<0.01***	*0.63*

AB, aortic banding; hypertrophy; HF, heart failure; IVSd, interventricular septal end-diastolic dimension; LVDd, left ventricular diameter at end-diastole; LVDs, left ventricular diameter at end-systole; FS, fractional shortening; PWd, posterior wall dimension; LAD, left atrial diameter.

**Figure 2:**
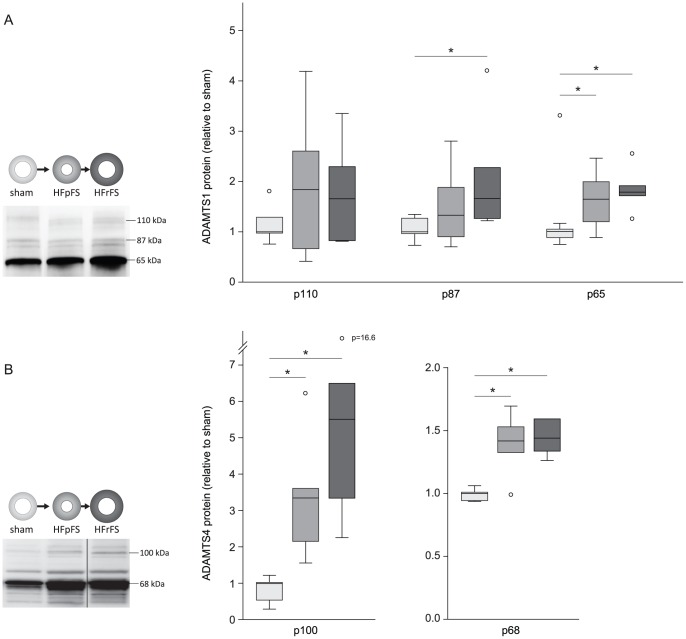
Increased protein expression of ADAMTS1 and -4 after aortic banding. Myocardial protein expression of ADAMTS1 (A) and -4 (B) in rats with preserved (HFpFS, n = 9) or reduced FS (HFrFS, n = 6) six weeks after aortic banding. Box-plots show median (horizontal line), interquartile range (box), 1.5xinterquartile range or maximum/minimum range (whiskers) and outliers (>1.5xinterquartile range). *p<0.05. AB, aortic banding; FS, fractional shortening.

Immunoblots of myocardial lysates from rats with AB were analyzed in order to evaluate protein levels of the proforms and mature forms of ADAMTS1 and −4 [27], [28]. In the HFrFS group, level of mature membrane-associated p87 form (1.66 (1.25–2.76)) and mature secreted p65 form (1.78 (1.59–2.06)) were all higher than in sham (p87: 1.00 (0.91–1.29), p = 0.03; p65: 1.00 (0.85–1.07), p<0.01) (Figure 2A). Furthermore, the p65 fragment expression was higher in HFpFS (1.64 (1.12–2.10) than in sham (p = 0.03), while no significant alterations was observed for the p110 proform (sham 1.00 (0.92–1.42, HFpFS 1.84 (0.60–3.00), HFrFS 1.66 (0.82-.56)). For ADAMTS4, the protein level of both the p100 proform and the mature membrane-associated p68 form was higher in the HFpFS (p100: 3.37 (2.02–4.30), p<0.01; p68: 1.41 (1.24–1.57), p = 0.03) and HFrFS group (p100: 5.55 (3.09–9.07), p<0.01; p68: 1.44 (1.32–1.59), p<0.01), than in sham (p100: 1.00 (0.48–1.08); p68: 1.00 (0.94–1.02)) (Figure 2B). The p53 form was not clearly visualized in the western blots.

### Myocardial mRNA levels of versican and aggrecan are increased in rats after AB

**Figure 3:**
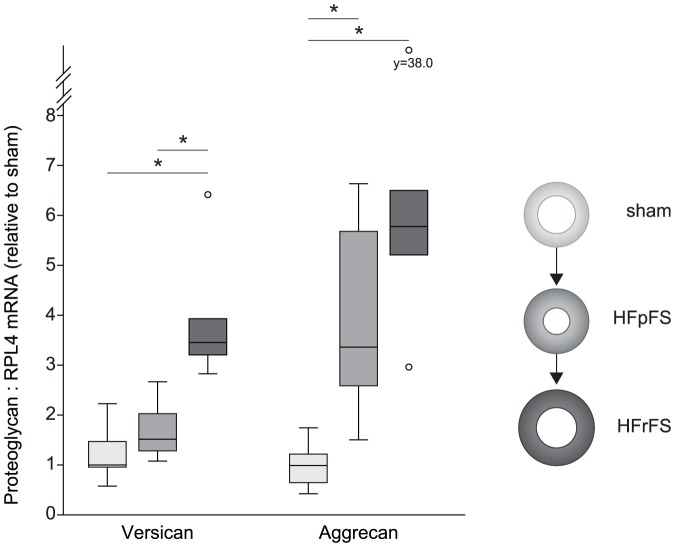
Elevated mRNA levels of versican and aggrecan in rats after aortic banding. Myocardial mRNA expression of versican and aggrecan in rats with preserved (HFpFS, n = 9) or reduced FS (HFrFS, n = 6) six weeks after AB, normalized to the reference gene RPL4 and relative to sham (n = 9). Box-plots show median (horizontal line), interquartile range (box), 1.5xinterquartile range or maximum/minimum range (whiskers) and outliers (>1.5xinterquartile range). *p<0.05. AB, aortic banding; FS, fractional shortening; ribosomal protein L4, RPL4.

Versican and aggrecan mRNA levels increased after AB (Figure 3). Compared to the versican level in sham (1.00 (0.92–1.55), a 3.5-fold increase in HFrFS (3.46 (3.11–4.58), p<0.01),and a tendency towards higher levels in HFpFS (1.49 (1.25–2.10, p = 0.06), were observed, in addition to a 2.3-fold increase in HFrFS compared to HFpFS (p<0.01). Similarly, the aggrecan level increased 4-fold in HFpFS (3.35 (2.55–5.98), p<0.01) and 6-fold in HFrFS (5.78 (4.65–14.38), p<0.01) compared to sham levels (1.00 (0.57–1.30). However, we did not observe any significant increase in aggrecan mRNA levels after transition to failure (HFpFS vs HFrFS: p = 0.16).

### Myocardial mRNA levels of MT4-MMP and HAS increased in rats after AB

**Figure 4:**
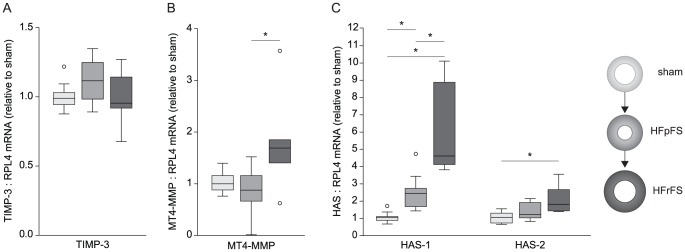
Myocardial TIMP-3, HAS and MT4-MMP mRNA levels in rats after aortic banding. Myocardial mRNA expression of TIMP-3, HAS and MT4-MMP in rats with preserved (HFpFS, n = 9) or reduced FS (HFrFS, n = 6) six weeks after aortic banding, normalized to the reference gene ribosomal protein L4 (RPL4) and relative to sham (n = 9). Box-plots show median (horizontal line), interquartile range (box), 1.5xinterquartile range or maximum/minimum range (whiskers) and outliers (>1.5xinterquartile range). *p<0.05. AB, aortic banding; FS, fractional shortening; TIMP, tissue inhibitor metalloprotease; HAS, hyaluronic acid synthase; MT, membrane-type.

Levels of TIMP-3, the endogenous inhibitor of ADAMTS4 [29], remained unchanged after AB (Figure 4A), whereas levels of MT4-MMP, which activates ADAMTS4 [28], were higher in HFrFS (1.69 (1.21–2.29) than in HFpFS (0.88 (0.57–1.23), p = 0.03) (Figure 4B). In HFrFS, we also found higher expressions of HAS-1 (4.55 (3.98–9.11)) and -2 (1.75 (1.37–2.82)) than in sham (HAS-1: 1.00 (0.75–1.18), p<0.01), HAS-2: 1.00 (0.66–1.33), p = 0.01) (Figure 4C).

### ADAMTS4 and versican levels were altered by pro-inflammatory mediators in cardiac cells

**Figure 5:**
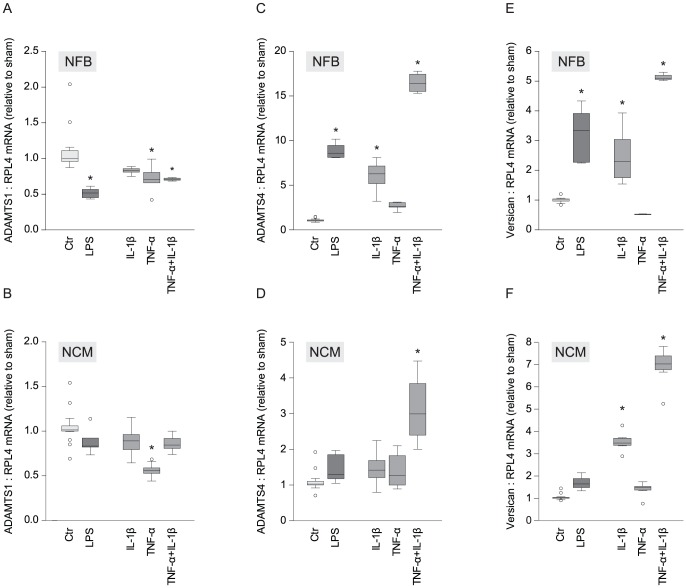
ADAMTS4 and versican mRNA in cardiac fibroblasts and cardiomyocytes was induced by inflammatory mediators. mRNA in NFB and NCM of ADAMTS1 (A,B), ADAMTS4 (C,D), and versican (E,F) after stimulation with LPS (n = 6), IL-1β (n = 7), TNF-α (n = 10 (NCM)/6 (NFB)), separate and combined (n = 7 (NCM)/4 (NFB)). Untreated cells served as control (n = 15 (NCM)/18 (NFB)). Box-plots show median (horizontal line), interquartile range (box), 1.5xinterquartile range or maximum/minimum range (whiskers) and outliers (>1.5xinterquartile range). *p<0.05, compared to control. NFB, neonatal fibroblasts; NCM, neonatal cardiomyocytes; LPS, lipopolysaccharide; IL, interleukin; TNF, tumor necrosis factor.

mRNA levels of versican and the two versicanases found increased in HFrFS-rats, namely ADAMTS1 and -4, were examined in NCM and NFB after treatment with inflammatory mediators. ADAMTS1 mRNA expression was lower in NFB treated with LPS, TNF-α alone and in combination with IL-1β (Figure 5A), and lower in TNF-α-treated NCM, than in control (Figure 5B). ADAMTS4 mRNA, on the other hand, increased after LPS- and IL-1β-treatment, and after combined stimulation with TNF-α and IL-1β in NFB (Figure 5C), while IL-1β and TNF-α in combination increased the ADAMTS4 level in NCM (Figure 5D). Similarly, versican levels increased in response to LPS, IL-1β alone and in combination with TNF-α in NFB (Figure 5E). In NCM, IL-1β alone and combined with TNF-α, induced enhancement in versican mRNA levels (Figure 5F).

**Figure 6:**
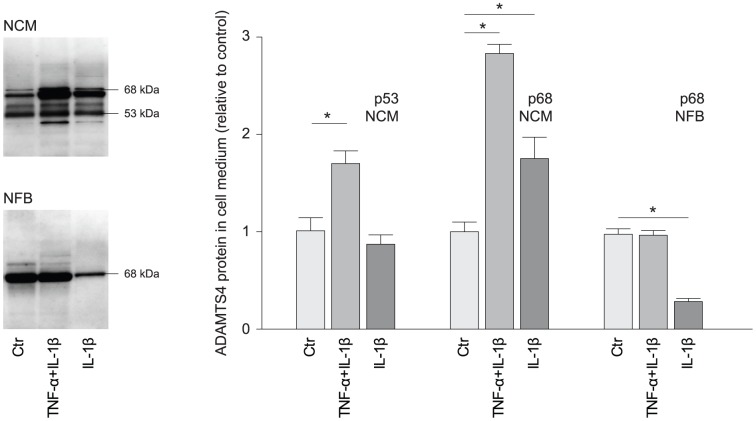
Altered ADAMTS4 protein levels in cell medium from cardiac cells after stimulation with inflammatory mediators. Protein levels of ADAMTS4 active forms (p68 and p53) in cell medium after stimulation of IL-1β alone (NFB n = 4, NCM n = 3) and in combination with TNF-α (NFB n = 3, NCM n = 4), relative to controls (n = 4). The bars represent mean levels and the error bars SEM. *p<0.05. NFB, neonatal fibroblasts; NCM, neonatal cardiomyocytes; LPS, lipopolysaccharide; IL, interleukin; TNF, tumor necrosis factor.

To assess secretion of active isoforms of ADAMTS4, we examined the protein level of ADAMTS4 in cell medium after stimulation with the identified inducers of mRNA synthesis, namely LPS, IL-1β alone and in combination with TNF-α. In NCM, enhanced levels of the mature membrane-associated p68 fragment was induced by IL-1β alone and in combination with TNF-α, while the mature secreted p53 fragment was induced by the combined stimulation of IL-1β and TNF-α only. The level of p68 isoform reduced in response to IL-1β alone (Figure 6). LPS reduced the level of p68 isoform in both cell types (data not shown). We did not identify the p53 isoform in NFB media.

### Improved heart function in AB-rats treated with PPS

Among the two ADAMTS versicanases, ADAMTS1 and -4, that were upregulated in HFrFS, we considered ADAMTS4 a better target for treatment due to the pronounced increase in HFrFS-rats, and inflammation-induced increase in cardiac cells. The semisynthetic polysaccharide PPS has known ADAMTS4 inhibitory effects [17], [18], and was thus chosen as an ADAMTS4-inhibitor for *in vivo* studies.

Interestingly, PPS-treated AB-rats demonstrated enhanced contractile function compared to vehicle-treated rats (Table 2). Briefly, we observed a 25% higher fractional shortening (FS), and a 33% lower left ventricular diameter in systole, in PPS-treated than in vehicle-treated AB-rats. Furthermore, a tendency towards lower lung weight in PPS-treated than in vehicle-treated AB-rats was observed (p = 0.13)

**Table 2:** Echocardiographic analyses and organ weights in PPS-treated rats after aortic banding.

	Sham vehicle	Sham PPS	*Sham vehicle vs sham PPS (p-value)*	AB vehicle	AB PPS	*AB vehicle vs AB PPS (p-value)*	*Sham vehicle vs AB vehicle (p-value)*
N	7	5		16	16		
ORGAN WEIGHTS							
Body (g)	398±13	408±18	*0.65*	374±8	363±6	*0.27*	*0.13*
Heart (g)	1.14±0.10	1.19±0.11	*0.74*	1.85±0.12	1.87±0.09	*0.88*	***<0.01***
LV (g)	0.66±0.02	0.70±0.03	*0.38*	0.98±0.04	1.00±0.04	*0.73*	***<0.01***
RV (g)	0.17±0.01	0.17±0.01	*0.89*	0.19±0.01	0.18±0.01	*0.80*	*0.38*
Lung (g)	1.37±0.04	1.41±0.03	*0.47*	1.96±0.19	1.64±0.08	*0.13*	*0.06*
M-MODE LV							
IVSd (mm)	1.50±0.03	1.56±0.04	*0.30*	2.05±0.07	2.11±0.06	*0.48*	***<0.01***
LVDd (mm)	6.70±0.25	6.79±0.14	*0.59*	6.73±0.13	6.52±0.13	*0.96*	*0.86*
LVDs (mm)	3.00±0.18	2.76±0.29	*0.36*	3.51±0.20	2.64±0.10	***<0.01***	*0.08*
FS (%)	55±2	59±4	*0.30*	48±3	60±1	***<0.01***	*0.08*
PWDd (mm)	1.57±0.03	1.68±0.08	*0.18*	2.05±0.05	2.13±0.06	*0.29*	***<0.01***
M-MODE AO/LA							
LAD (mm)	3.55±0.08	3.68±0.14	*0.39*	4.29±0.19	4.18±0.14	*0.64*	***0.02***
Aorta (mm)	2.64±0.05	2.63±0.04	*0.88*	2.52±0.03	2.56±0.03	*0.30*	***0.02***
DOPPLER							
Gradient day 3	-	-	*-*	4856±221	4796±177	*0.83*	***-***
Peak mitral flow (m/s)	939±40	946±45	*0.92*	1088±27	1069±40	*0.70*	***<0.01***
Mitral deceleration (m/s)	2710±255	2368±388	*0.46*	3877±333	4415±388	*0.30*	***0.04***
Heart rate	437±24	412±11	*0.42*	405±8	420±6	*0.14*	*0.12*
TISSUE DOPPLER							
Maximal systolic velocity	74±5	73±9	*0.88*	53±3	51±3	*0.68*	***<0.01***
Maximal diastolic velocity	80±4	82±6	*0.72*	55±3	59±3	*0.34*	***<0.01***

AB, aortic banding; LV, left ventricle; IVSd, interventricular septal thickness at diastole; LVDd, left ventricular diameter at diastole; LVDs, left ventricular diameter at systole; FS, fractional shortening; PWDd, posterior wall thickness at diastole; LAD, left atrial diameter; RV, right ventricle.

However, no significant alterations were observed for diastolic function, including mitral deceleration, peak mitral flow, left atrial diameter, or maximal diastolic velocity. Parameters for hypertrophy was not affected either by PPS-treatment in rats after AB (Table 2). The mortality rate was similar in both treatment groups (vehicle: 8/24 vs PPS: 6/22, p = 0.66).

### PPS-treatment reduced myocardial ADAMTS4 mRNA levels

**Figure 7:**
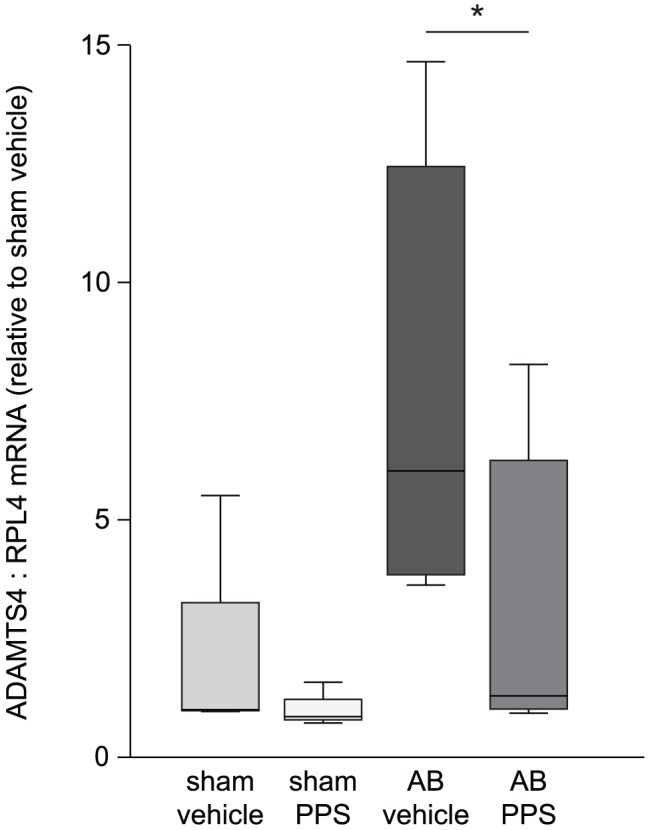
Decreased myocardial ADAMTS4 mRNA level after PPS-treatment. Myocardial mRNA expression of ADAMTS4 in rats treated with PPS (n = 10) or vehicle (n = 7) six weeks after AB, and in sham-operated rats treated with PPS (n = 3) normalized to the reference gene RPL4 and relative to vehicle-treated sham (n = 3). Box-plots show median (horizontal line), interquartile range (box), 1.5xinterquartile range or maximum/minimum range (whiskers) and outliers (>1.5xinterquartile range). *p<0.05. AB, aortic banding; PPS, pentosan polysulfate; HF, heart failure; RPL4, ribosomal protein L4.

**Figure 8:**
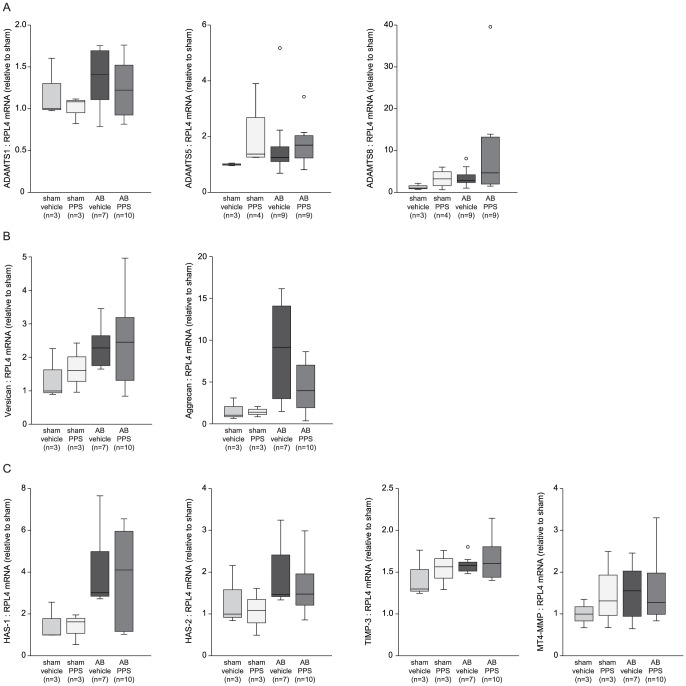
Myocardial mRNA levels after PPS treatment in rats after aortic banding. Myocardial mRNA expression of ADAMTS1, -5, -8 (A), versican, aggrecan (B), HAS-1, -2, TIMP-3, and MT4-MMP (C) in rats treated with PPS (n = 9 or 10) or vehicle (n = 7 or 9) six weeks after AB, and sham-operated rats treated with PPS (n = 3 or 4) normalized to the reference gene ribosomal protein L4 (RPL4) and relative to vehicle-treated sham (n = 3). The bars represent median levels and the error bars the 75^th^ percentile. *p<0.05. HF, heart failure, HA, hyaluronic acid.

ADAMTS4 mRNA level was reduced in AB-rats treated with PPS (1.29 (1.00–6.45)) compared to vehicle-treated AB-rats (6.03 (3.79–12.76), p = 0.03) (Figure 7). On the contrary, PPS-treatment did not alter the mRNA levels of versican (vehicle 2.28 (1.67–2.77), PPS 2.46 (1.31–3.23), p = 0.85), or the versicanases ADAMTS1 (vehicle 1.41 (1.10–1.72), PPS 1.22 (0.91–1.58), p = 0.70) or -5 (vehicle 1.26 (1.04–1.94), PPS 1.70 (1.24–2.10), p = 0.40). Neither the mRNA levels of aggrecan (vehicle 9.10 (1.69–14.84), PPS 3.93 (1.71–7.30), p = 0.17) nor the aggrecanase ADAMTS8 (vehicle 2.77 (1.87-5-12), PPS 4.63 (1.74–13.52), p = 0.27) were affected by PPS treatment (Figure 8).

PPS treatment did not affect mRNA levels of proteins known to modulate versican or ADAMTS4 activity, namely HAS-1 (ctr 3.02 (2.79–5.60), PPS 2.70 (0.75–4.07), p = 0.77), HAS-2 (ctr 1.47 (1.39–2.52), PPS 1.11 (0.85–1.59), p = 0.38), TIMP-3 ((ctr 1.35 (1.23–1.44), PPS 1.09 (0.87–1.42), p = 0.92), or MT4-MMP levels ((ctr 1.56 (0.93–2.15), PPS 1.27 (0.99–2.05), p = 0.83) (Figure 8).

### PPS-treatment inhibited myocardial versican cleavage in AB-rats

**Figure 9:**
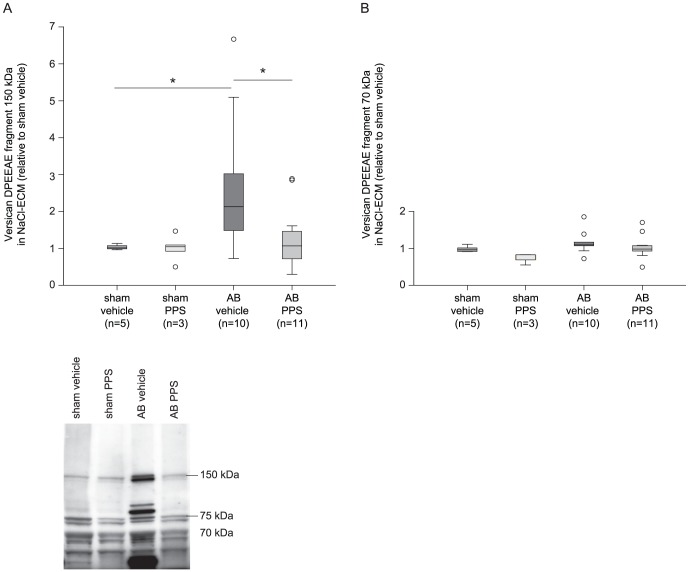
Versican p150 fragment level was reduced after PPS-treatment in rats with aortic banding. Myocardial levels of p150 (A) and p70 (B) versican DPEEAE fragment in NaCl-soluble ECM of rats subjected to AB with or without PPS-treatment, shown as representative immunoblot and box-plots with median (horizontal line), interquartile range (box), 1.5xinterquartile range or maximum/minimum range (whiskers) and outliers (>1.5xinterquartile range). *p<0.05. AB, aortic banding; PPS, pentosan polysulfate; ECM, extracellular matrix; HF, heart failure.

Presence of versican fragmentation was evaluated by immunoblotting using an antibody detecting the neo-epitope DPEAAE displayed after cleavage of versican by ADAMTS1/4, where fragments of 150 and 70 kDa result from cleavage of the V0 and V1 isoform, respectively [25], [26]. In NaCl-ECM, we observed a higher amount of p150 versican in vehicle-treated AB (2.14 (1.34–3.55)) than in vehicle-treated sham (1.06 (0.73–1.30), p = 0.04). Following PPS-treatment, the levels of p150 versican in NaCl-ECM were reduced by ∼50% (1.08 (0.69–1.63)) compared to vehicle-treated AB-rats (p = 0.05). A tendency towards higher amounts of the p70-fragment in NaCl-ECM in vehicle-treated AB-rats (1.08 (1.02–1.21) than in both PPS-treated AB-rats (0.97 (0.90–1.07), p = 0.14) and vehicle-treated sham rats (0.95 (0.90–1.05), p = 0.11) were observed (Figure 9).

## Discussion

In this study, we discovered that PPS-treatment improved systolic function in the pressure -overloaded heart. Our findings suggest that this beneficial effect of PPS was mediated through a reduction in ADAMTS4 versicanase activity, based on the following main findings: 1) increased ADAMTS4 expression and altered ADAMTS-induced versican degradation during heart failure development, 2) inflammation-induced ADAMTS4 and versican synthesis in cardiac cells, and 3) improved contractile performance accompanied by indications of reduced ADAMTS4 versicanase activity in AB-rats receiving PPS. In summary, our findings shed light on a novel mechanism for heart failure development, and a promising novel heart failure therapy.

### Enhanced ADAMTS activity during heart failure development

To the best of our knowledge, we are the first to report increased synthesis and activity of ADAMTS versicanases and aggrecanases during heart failure progression. First, in the myocardium of AB-rats with reduced FS, an increase in mRNA synthesis of ADAMTS1, -4 and -8, and enhanced protein levels of ADAMTS1 and -4, was observed, concurrent with an increase in the ADAMTS4 activating enzyme MT4-MMP. In addition, mRNA synthesis of versican and aggrecan increased, providing substrate for ADAMTS-mediated fragmentation.

Synthesis of ADAMTS4 and versican in cardiac cells was induced *in vitro* by TNF-α and IL-1β, cytokines found in enhanced levels in the failing myocardium [15], [16], suggesting inflammation as a trigger for ADAMTS-mediated versican fragmentation in heart failure. Secretion of active isoforms of ADAMTS4 was also enhanced in cardiomyocytes after combined stimulation of IL-1β and TNF-α, whereas the increases in cellular levels in fibroblasts were not followed by increased secretion of the enzyme. Nevertheless, we regard both cardiomyocytes and fibroblasts as potential sources for ADAMTS4, and believe that our findings in cell culture reflect a sophisticated regulation of ADAMTS4 production and activation, involving cell-specific and synergistic effects within a complex cytokine milieu that are difficult to reproduce in vitro. Indeed, the post-translational processing of ADAMTS4 is known to involve several steps and modulatory factors; the activation of p68 isoform through prodomain removal and activation of p53 isoform through C-truncation is mediated by furin and MT4-MMP, respectively, while several other mediators, like TIMP, may also modulate processing [28], enabling distinct regulation of the various processing steps.

The notion of accelerated ADAMTS-induced versicanase activity in response to pressure overload was supported by increased amounts of versican p150 DPEEAE fragments resulting from ADAMTS1/4-induced cleavage. After AB, the amount of versican p150 fragment doubled in NaCl-ECM, the fluid-compartment of the ECM. Interestingly, ADAMTS4 lacks the thrombospondin repeat tail that links other ADAMTS proteases to ECM components [30], suggesting that ADAMTS4 is located in the NaCl-soluble ECM. Taken together, the observed accumulation of p150 versican fragments is probably caused, at least partially, by an accelerated ADAMTS4 activity.

### PPS-treatment reduced myocardial ADAMTS4 versicanase activity

PPS-treatment of AB-rats counteracted several of the alterations in ADAMTS4 and versican fragment levels observed in vehicle-treated AB-rats. While previous studies have demonstrated that PPS inhibits aggrecanase activity of ADAMTS4 and -5 [17] without affecting gene expression of the protease [18], we did observe reduced mRNA levels of ADAMTS4 after PPS-treatment. However, reduced expression of ADAMTS5 has been observed in PPS-treated rats [22], supporting that inhibitory actions of PPS also can be mediated through reduced enzyme expression. Interestingly, the level of TIMP-3 was not affected, resulting in a net increase of the protease to inhibitor ratio. Indeed, downregulation of p150 DPEEAE versican fragments in NaCl-ECM in PPS-treated AB-rats supports that PPS inhibit ADAMTS4 versicanase activity in the pressure-overloaded heart.

### PPS-treatment improved contractile function in AB-rats

PPS-treatment improved contractile function in rats after AB, potentially halting the critical phase of transition to failure. The concurrent reduction in ADAMTS4 expression and amount of versican p150 fragments observed after PPS-treatment, suggests an association between versican degradation and deterioration of systolic function. Versican enables cartilage to resist compression by reducing fluid movements [31], and its viscoelastic properties are likely to be important also in myocardial mechanics regarding the repetitive cycles of contraction and relaxation. The accumulation of versican p150 fragments in the NaCl-ECM observed in our study could reflect an unfavorable increase in myocardial water content, at least in some compartments of the ECM. Indeed, heart failure is associated with myocardial edema [32], and enhanced interstitial hydration may compromise systolic function and attenuate oxygen delivery [33]–[35]. Interestingly, swelling of ECM due to versican accumulation exerts detrimental effects in other organs; breakdown of versican in lungs worsen lung edema [36], while accumulation of versican contributes to the increased ECM volume that occurs during formation of atherosclerotic plaques [37]. In addition, degradation of versican may also potentially disrupt the proteoglycan-rich endothelial glycocalyx, resulting in increased vascular permeability and myocardial edema [38], [39]. However, the contribution of versican degradation to myocardial mechanics remains insufficiently explored and merits further elucidation in future studies.

As opposed to collagens, proteoglycans provide limited tensile strength [40] and their contribution to passive stiffness could therefore be minor, thus supporting the lack of diastolic alterations in PPS-treated animals. Unknown extracardiac effects of PPS potentially affecting afterload may also serve as an explanation to the selective effective on systolic function. Taken together, PPS-treatment seems to enhance systolic function by modulating myocardial mechanics in a manner that maintains diastolic function.

### Clinical implications

ADAMTS4 represents a promising novel therapeutic target in patients with aortic stenosis. PPS has FDA-approval, and is used in treatment of interstitial cystitis patients and osteoarthritis in veterinary medicine. While PPS reduces infarction size in a reperfusion model [41], its potential as a target in chronic heart failure has not previously been explored. PPS is well tolerated and besides a mild anti-coagulative effect, PPS exerts few side effects [42]. Thus, PPS holds promise as a novel therapeutic agent in heart failure.

### Limitations of the study

Further studies are needed to establish the cause-effect relationship between PPS, ADAMTS4-inhibition and improved contractile performance. Due to the lack of opportunity to subdivide AB-rats into the failing and non-failing phenotype during *in vivo* ADAMTS4-inhibition, the rats with AB receiving vehicle- or PPS-treatment should be regarded as a group consisting of both phenotypes. Although PPS-treatment led to a significant improvement in parameters for systolic function, the heterogeneity within the group regarding heart failure development could render the study underpowered to detect other pathophysiological characteristics, such as left atrial diameter and lung weight. Studying the effects of PPS-treatment at different time-points after induction of pressure overload may reveal effects on these pathophysiological characteristics, but is beyond the scope of this study. Furthermore, ADAMTS4-independent effects of PPS need to be explored in further detail in future studies. PPS also suppresses the activity of ADAMTS5 [17], inflammatory mediators, and coagulation factors [41], effects that may contribute to the observed beneficial effect in the pressure-overloaded heart. The role of aggrecanase activity exerted by ADAMTS1, -4, and -8 in heart failure progression will hopefully also be addressed in studies to come.
